# An Unusual Dimeric Inhibitor of Acetylcholinesterase: Cooperative Binding of Crystal Violet

**DOI:** 10.3390/molecules22091433

**Published:** 2017-08-30

**Authors:** Anders Allgardsson, C. David Andersson, Christine Akfur, Franz Worek, Anna Linusson, Fredrik Ekström

**Affiliations:** 1 Swedish Defence Research Agency, SE-90281 Umeå, Sweden; anders.allgardsson@foi.se (A.A.); christine.akfur@foi.se (C.A.); 2 Department of Chemistry, Umeå University, SE-90187 Umeå, Sweden; david.c.andersson@umu.se (C.D.A.); anna.linusson@umu.se (A.L.); 3 Department of Toxicological Enzymology, Bundeswehr Institute of Pharmacology and Toxicology, 80937 Munich, Germany; franzworek@bundeswehr.org

**Keywords:** cholinesterase, acetylcholinesterase, cooperativity, crystal violet, Hill coefficient, new modality, non-bonded bivalence

## Abstract

Acetylcholinesterase (AChE) is an essential enzyme that terminates cholinergic transmission by a rapid hydrolysis of the neurotransmitter acetylcholine. AChE is an important target for treatment of various cholinergic deficiencies, including Alzheimer’s disease and myasthenia gravis. In a previous high throughput screening campaign, we identified the dye crystal violet (CV) as an inhibitor of AChE. Herein, we show that CV displays a significant cooperativity for binding to AChE, and the molecular basis for this observation has been investigated by X-ray crystallography. Two monomers of CV bind to residues at the entrance of the active site gorge of the enzyme. Notably, the two CV molecules have extensive intermolecular contacts with each other and with AChE. Computational analyses show that the observed CV dimer is not stable in solution, suggesting the sequential binding of two monomers. Guided by the structural analysis, we designed a set of single site substitutions, and investigated their effect on the binding of CV. Only moderate effects on the binding and the cooperativity were observed, suggesting a robustness in the interaction between CV and AChE. Taken together, we propose that the dimeric cooperative binding is due to a rare combination of chemical and structural properties of both CV and the AChE molecule itself.

## 1. Introduction

The enzyme acetylcholinesterase (AChE) catalyses the hydrolysis of the neurotransmitter acetylcholine, and is an essential component of the central and peripheral nervous system. The pioneering work that led to the first atomic resolution structure of AChE revealed a deep, sterically confined, and highly aromatic active site gorge [1]. The structure proved a milestone in protein research and has inspired numerous studies of the relation between the structure and dynamics of the active site gorge, and the functional properties of AChE. The molecular recognition of AChE is intricate and typically involves multiple transient binding sites, aromatic interactions, or non-conventional hydrogen bonds [2,3,4,5]. Due to the pivotal role in cholinergic transmission, AChE is an important target for synthetic drugs used for symptomatic treatment of Alzheimer’s disease [6,7], for antidotes used to counteract organophosphorus compounds (i.e., pesticides and nerve agents) [8], and for insecticides used to control vector borne diseases [9].

We have previously reported the in vitro screening campaigns of a chemically diverse library consisting of 17,500 drug-like compounds. The screens were performed using AChEs derived from *Homo sapiens* (*h*AChE), *Anopheles gambiae* (*Ag*AChE), and *Aedis aegypti* (*Aa*AChE), and collectively led to the discovery of 425 unique hits that significantly reduced the enzymatic activity at a concentration of 50 µM [10,11]. Subsequent hit confirmation resulted in the determination of the half maximal inhibitory concentration values (IC_50_) and Hill coefficients (*n*_H_) for 106 compounds. In biochemistry and pharmacology, the Hill coefficient is commonly used for determining the degree of cooperativity of a ligand binding to a target [12]. The concept of cooperative ligand binding most commonly refers to multimeric, multivalent proteins, where the probability of ligand binding will be increased (positive cooperativity) or decreased (negative cooperativity) if one or several of the ligand binding sites are already occupied [12]. A classic example is haemoglobin, where Christian Bohr already in 1904 found that the more oxygen that is bound to haemoglobin, the easier it is for additional oxygen to bind [13]. Cooperativity has since then been observed in a wide variety of biological macromolecules including enzymes [14], ion channels [15,16], and transcription factors [17].

**Figure 1:**
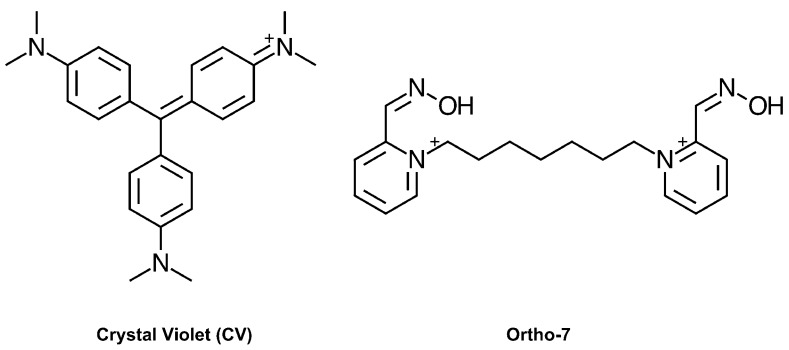
Chemical structure of crystal violet (CV) and Ortho-7.

While the hits identified in our screen displayed a considerable diversity regarding size, polarity, flexibility, charge distribution, and potency, their Hill coefficients typically approached 1, which is consistent with non-cooperative binding. One surprising exception was the dye crystal violet (CV, Figure 1), which displayed an unusually high Hill coefficient (*n*_H_ > 1) that warranted further investigation. The antibacterial activity of CV is well established [18], and dyes based on a hexamethylparaosaniline scaffold have previously been reported to inhibit AChE [19], and the nicotinic acetylcholine receptor [20], although with no details of the mode of binding. To elucidate the molecular basis for this observation, we herein report the combined use of kinetics, site directed mutagenesis, X-ray crystallography, and density functional theory (DFT) calculations.

## 2. Results

### 2.1. Kinetic Support of Positive Cooperative Inhibition

**Figure 2:**
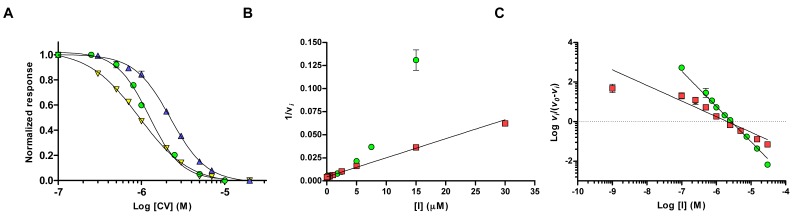
Dose-response (**A**) of CV binding to *h*AChE (green circles), *m*AChE (blue triangles) and *h*BuChE (yellow triangles). Dixon plot (**B**) and Hill plot (**C**) of CV (green circles) and Ortho-7 (red squares). In the figure, *v_o_* is the rate without any inhibitor and *v_i_* is the rate in presence of various concentrations of the inhibitor.

To confirm the high *n*_H_ observed during the hit confirmation experiments [10], we started by determining the IC_50_ values of CV by using wild-type *h*AChE, *Mus musculus* AChE (*m*AChE), and *Homo sapiens* butyrylcholinesterase (*h*BuChE). The dose-response curves were analyzed using a four parameters logistic (4PL) model, with a variable slope that allows non-linear fitting of the Hill coefficient. These experiments showed that the half maximal inhibition concentration (IC_50_) was 2.2 μM, and 1.2 μM for *m*AChE, and *h*AChE, respectively (Table 1, Figure 2A). Moreover, the Hill coefficients were −2.0 (*m*AChE) and −2.2 (*h*AChE), consistent with positive cooperative inhibition. Note that the negative values of *n*_H_ was because we did not directly measure the binding of CV, but rather the decrease in hydrolysis of the substrate analogue acetylthiocholine iodide (AChI). The corresponding analyses of binding to *h*BuChE revealed a slightly lower IC_50_ value of 0.9 μM, and a significantly lower Hill coefficient of −1.4 (Table 1, Figure 2A), pointing at a less cooperative binding of CV to *h*BuChE.

In an effort to determine the inhibition constant (*K_i_*), and to establish the mode of inhibition, the apparent Michaelis–Menten constants (*K_M_^app^*), and the apparent maximal velocities (*V_max_^app^*) were determined at different concentrations of CV. Typical re-plots such as 1/*V_max_^app^* and *K_M_^app^*/*V_app_* versus [CV] showed a non-linear behavior, which precluded further analysis of the data.

A third set of experiments was performed at a substrate concentration of 100 μM (~75% of the Michaelis-Menten constant, *K_M_*), where the non-linear parabolic behavior is evident in the Dixon plot (Figure 2B). As a comparison, the Dixon plot of 1,7-heptylene-bis-*N*,*N*′-2-pyridiniumaldoxime dichloride (Ortho-7, Figure 1) can be fitted by linear regression (r^2^ of 0.98) under these conditions (Figure 2B). Moreover, IC_50_ determination of Ortho-7 shows an IC_50_ of 2.1 μM, and a *n*_H_ of −1.1, in agreement with the monomeric binding site of Ortho-7, as revealed by crystallographic studies [21]. Further analysis of the data using a Hill plot show distinctively different slopes of −1.8 and −0.8 for CV and Ortho-7, respectively (Figure 2C), in agreement with the IC_50_ determinations. The dissociation constant (*K_d_*) at the intercept with the *X*-axis is 2.7 and 2.1 μM, for CV and Ortho-7, respectively. Taken together, these experiments confirm that inhibition of *h*AChE and *m*AChE by CV displays a significant positive cooperativity with an *n*_H_ of 2.0–2.2. As the corresponding experiments using *h*BuChE have an *n*_H_ of 1.4, we also conclude that the effect is linked to the specific binding of CV to AChE, and not an intrinsic property of the CV molecule itself.

### 2.2. X-ray Crystal Structure of Crystal Violet in Complex with Mus musculus AChE Establish Structural Basis of Cooperativity

**Figure 3:**
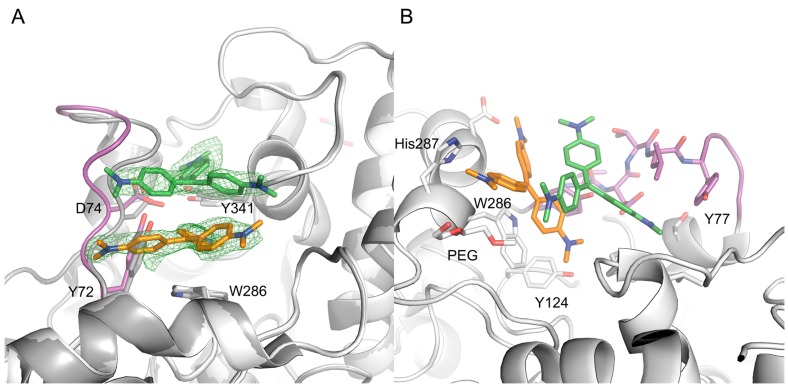
The electron density map clearly defines CV1 (orange) and CV2 (green) binding at the entrance of the active site gorge (**A**); The binding of CV1 and CV2 involves an extensive interface, with high shape complementarity (**B**). In the picture, the 72–80 loop region is shown in magenta, and the apo form of AChE is shown in dark grey.

To investigate the binding of CV, we determined the X-ray crystal structure of a complex between CV and *m*AChE, to a resolution of 2.4 Å (see Appendix, Table A1). Overall, the structure of the complex is similar to the structure of apo *m*AChE (PDB (Protein Data Bank) entry code 1J06). However, as a consequence of the ligand binding, a significant shift of up to ~3 Å of the protein backbone/main chain in the loop region comprising residues Tyr72 to Phe80 is evident (Figure 3A).

The initial, unbiased Fo-Fc electron density map clearly defines the two CV molecules binding at the entrance to the active site gorge of each AChE monomer (Figure 3A). Traces of a third CV molecule is visible at a contour level close to the noise of the electron density maps, but the molecule was not included in the refinements. The two CV molecules (denoted CV1 and CV2) form a dimer that effectively blocks access to the active site. The dimer is not linked via covalent bonds (i.e., non-bonded). The final refinement involved the use of DFT (B3LYP/6-31G**) geometry optimized structures to model the dihedral angles between the aryl groups and the central coordination plane. The bound CV molecules each adopt a propeller-like geometry adopting the D3 point group symmetry, as previously determined by Raman spectroscopy [22,23] and small molecule X-ray crystallography [24]. Furthermore, the arenes are tilted with respect to the central coordination plane, and the dimethylamino group of each arene is nearly in plane with its ring. The stability of the CV dimer in the absence of mAChE was investigated using DFT. Geometry optimizations of the dimer in water led to negative vibrational frequencies indicative of a non-optimal structure. In the gas phase, the dimer dissociated completely (B3LYP/6-31G**) or showed a positive interaction energy of 11 and 20 kcal mol^−1^ in gas the phase using methods BLYP-D3/6-31G** and M06-2X/6-31G**, respectively, indicating that the dimer is energetically unfavorable outside of *m*AChE.

**Figure 4:**
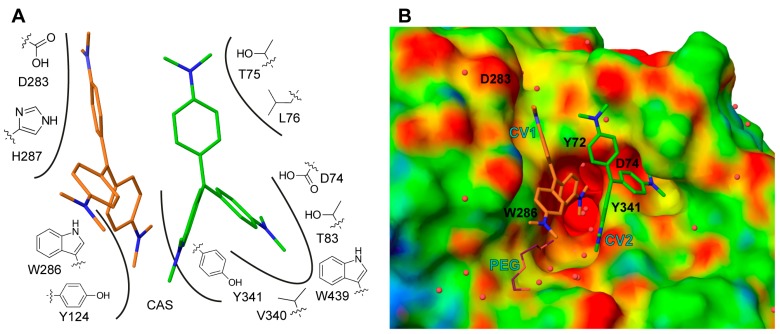
Schematic (**A**) of the interactions between CV1 (orange), CV2 (green) and *m*AChE, where interacting amino acids are indicated with number and is the catalytic site (CAS). *m*AChE electrostatic potential surface (**B**) with low, medium and high potential coloured in red, green and blues, respectively. The positions of mutated amino acids are indicated with residue name and number. For clarity, the polyethylene glycol (PEG) molecule is omitted in A.

CV1 is positioned close to the α-helix spanning residues Ala278 to Val288 at the entrance of the active site gorge, with one dimethylaminobenzene moiety directed towards the catalytic site (CAS). CV1 interacts with the protein through an extensive interface involving arene-arene interactions, electrostatic interactions, and van der Waals contacts with residues Tyr72, Tyr124, Val282, Asp283, Trp286, His287, and Tyr341 (Figure 4A). In addition, one of the dimethylamino groups of CV1 interacts with a polyethylene glycol molecule; polyethylene glycol (PEG) is used as a precipitant during crystallization (Figure 4B). Apparently, the CV1–AChE complex forms a surface that allows for the binding of CV2. In this, both CV1 and the side chains of residues Tyr72, Asp74, Thr75 Leu76, Tyr77, Thr83, Val340 W439, and Tyr341 forms the pocket that accommodates CV2 (Figure 4A,B). Taken together, the structural analysis unambiguously maps the binding site of two CV monomers that have an excellent shape complementarity to AChE (Figure 4B), and effectively block access to the active site gorge.

### 2.3. Site Directed Mutants Probing the Binding Site of CV

To investigate the specific contributions of residues comprising of the interface between AChE and the nonbonded dimer of CV, we introduced site directed substitutions in positions surrounding the binding site of CV1 or CV2 (Figure 4B). The mutagenesis focused on changes of the electrostatics (at position 74 and 283) or reducing aromaticity (at position 72, 286 and 341), and was performed using both *h*AChE (Y72A, D74A, D74N, N283A, N283D, W286A, and Y341A) and *m*AChE (D283A and D283N) as a template. The IC_50_ values and Hill coefficients were subsequently determined using the 4PL model (Table 2). We found that the single sites mutants caused a limited three- to four-fold increase of the IC_50_ values. The most pronounced effect was observed for the D283A mutant of *m*AChE and for D74A, D74N, and W286A substitutions of *h*AChE. The moderate effect on the IC_50_ of these substations is somewhat surprising. As a comparison, the binding of the reversible peptide inhibitor fasciculin from the green mamba (*Dendroaspis angusticeps*), is highly dependent on interactions with the aromatic residues Tyr72, Tyr124, Trp286, and Tyr341 [25,26].

The substitutions had a noticeable effect on the cooperativity and the Hill coefficients ranged from −1.5 (for D283A of *m*AChE) to −2.1 for W286A *h*AChE. It should be noted that the Hill coefficient describes the degree of cooperativity, and the value of *n*_H_ is not necessarily the same as the stoichiometry between CV and AChE [27]. In general, substitutions that affected the electrostatic properties had a larger effect on the *n*_H_ than mutants that reduced the aromaticity. The data showed no correlation between IC_50_ and *n*_H_. To investigate if the potential role of global, long-range forces, such as the dipole of AChE, contribute to the binding of the cationic CV molecules, we calculated the dipole moment of wild type and mutant AChEs, as well as of *h*BuChE (Table 3). The dipole moment for wild-type *h*AChE and *m*AChE was in the range of 600–700 Debye, and the mutations did not change the dipole moment significantly. Interestingly, the dipole moment of *h*BuChE (*n*_H_ = −1.4 and IC_50_ = 0.9 µM) and *Torpedo californica* AChE (*Tc*AChE) was substantially larger (~1700 Debye). We conclude that the effect on CV binding and cooperativity seen for the mutants cannot straightforwardly be correlated to the overall dipole moment of AChE.

Taken together, the experiments suggest that the binding is driven by multiple intermolecular contacts, and that single point mutations are insufficient to disturb the interaction. The data also infer that the interaction forces between AChE and CV are robust and lack directionality that limits the implications of the structural distortions induced by the mutagenesis.

## 3. Discussion and Conclusions

The fold, molecular architecture, and ligand binding properties of AChE is well studied with over 200 crystal structures deposited in the PDB [28]. For numerous reversible ligands, dose-response curves are also available. A recent study of the inhibitor hopeahainol shows an inhibition kinetics that is consistent with cooperative binding to AChE [29]. Still, cooperativity appears rare, and is linked to the specific chemistry and structure of the CV•AChE complex and is not a common, inherent property of cholinesterases. We also conclude that the cooperativity is strong and depends on the specific interaction between CV and AChEs, and not on the CV molecules themselves. For example, the *n*_H_ is approximately −2.0 for AChEs and −1.4 for *h*BuChE, despite similar IC_50_ values. Furthermore, the D283A substitution of *m*AChE reduced the *n*_H_ from −2.0 for wild type to −1.5 for the mutant, clearly showing the importance of the CV–AChE interface.

The X-ray crystal structure of CV in complex with *m*AChE, revealed a unique binding pose where two monomers of CV bind to a pocket close to the entrance of the active site gorge. The structure shows that the monomers form extensive inter-molecular contacts between each other and to the protein. The crystal structure also shows a significant structural change of the loop between residue Tyr72 and Phe80. Based on the structure, we conclude that the cooperativity is homotropic (i.e., occur via the binding of two chemically identical ligands), and that the inhibition of AChE is due to steric blocking of the entrance of the active site gorge. We subsequently used a computational approach to investigate the stability of the CV dimer in solution. Our calculations suggest that the dimer is not stable in solution suggesting that the protein environment is necessary for dimerization of CV. The crystal structures of CV in complex with the *Staphylococcus aureus* multidrug binding protein QacR [30], and the complex with the multidrug-resistance regulator RamR have been reported [31]. In both of these cases, the CV molecule is monomeric. Together with our computational analysis, this strongly supports a sequential binding of two CV monomers. Further mechanistic studies using more complex kinetic models may provide separated binding constants of the binding events.

Ligands that form dimers with extensive intermolecular contacts that facilitate their association and binding to the target have been reported earlier. For example, polyamines form dimers that bind to the minor groove of double helical B-DNA [32]. Also in this case, a computational study supported that the polyamines exist as monomers in solution, and it was suggested that ligands that dimerize may offer opportunities to improve affinity, selectivity, and cell permeability of organic ligands [33]. It should be noted that ligands that forms dimers in their binding site do not necessarily display cooperative binding. While both cooperativity and non-bonded dimeric ligands have been independently reported previously, the AChE–CV system is to the best of our knowledge the first example where the two phenomena have been linked and described in a single system. While the utility of this new concept in drug discovery and medicinal chemistry remains to be investigated, the principle may offer novel opportunities to use small organic molecules to modulate challenging targets, such as protein–protein or protein–nucleic acid interactions.

## 4. Methods

### 4.1. Expression, Site Directed Mutagenesis and Determination of Kinetic Parameters

Recombinant *m*AChE and *h*AChE was expressed as previously described [34], whereas *h*BuChE was purchased from Sigma-Aldrich (Darmstadt, Germany). Site directed mutagenesis of *h*AChE was performed as reported [35]. The enzymatic activity of AChE and *h*BuChE was measured spectrophotometrically using a method previously reported [36]. All assays were performed at 30 °C in 0.1 M phosphate buffer at pH 8.0 using acetylthiocholine iodide (AChI) or buturylthiocholine iodide (BuChI) as a synthetic substrate. CV (tris(4-(dimethylamino)phenyl)methylium chloride) was supplied by Sigma-Aldrich (USP testing grade). Initial rates were determined using a plate reader (Biotek powerwave, Biotek, Winooski, VT, USA) or an UV/Vis spectrophotometer (Perkin Elmer Lambda 650, Perkin Elmer, Waltham, MA, USA). The substrate concentration was 1 mM for the IC_50_ determinations, 0.02–0.8 mM for the determination of *K_i_*, and 0.1 mM for the data used for the Hill-plot. Analysis of data was performed using four parameters (variable slope) non-linear regression using GraphPad Prism version 5 for Windows (GraphPad Software, La Jolla, CA, USA).

### 4.2. Purification, Crystallization and Structure Determination

Expressed AChEs were purified and crystallized as previously reported [35,37]. The crystallized ternary complexes between *m*AChE and CV were generated by a stepwise addition of a saturated ligand soaking solution (consisting of 30% (*v*/*v*) polyethylene glycol 750 monomethylether, 100 mM HEPES, pH 7.1) to crystals of *m*AChE that, after incubation for an additional 10 minutes, were vitrified in liquid nitrogen. X-ray diffraction data were collected at beam-line I911-3 at the MAX-lab synchrotron (Lund, Sweden) on a MAR Research CCD detector, using an oscillation angle of 1.0° per exposure. The intensity data were indexed and integrated using XDS, and scaled using Scala [38,39]. The initial model was determined using difference Fourier methods using a native *m*AChE structure (PDB entry code 1J06). The identification and placement of the ligands were based on the initial 2|F_O_| − |F_C_| and |F_O_| − |F_C_| maps. Automated and manual model refinement was then performed using the Phenix software suite and COOT [40,41]. In order to ensure proper ligand geometries, DFT geometry optimization was included in the refinement protocol as previously reported [4,42,43]. The structure factors and refined coordinates have been deposited in the Protein Data Bank, accession code 5OV9.

### 4.3. Density Functional Theory Calculations

Monomers and dimers of CV (1+) were geometry optimized by using the basis set 6-31G** and functionals B3LYP [44,45,46], M06-2X [47], and BLYP-D3 [48] in gas phase, and using a Poisson-Bolzmann water model implemented in Jaguar version 9.1 (Schrödinger, Inc., New York, NY, USA) [49]. CV was considered as a cation in all calculations. Geometry optimizations were run until conversion by using the direct inversion of the iterative subspace (DIIS) method, alternatively, until dimer dissociation. Interaction energies (ΔE) were calculated by the subtraction of energies of the monomer from that of the dimer.

### 4.4. Dipole Moment and Electrostatic Potential Calculations

Dipole moments were calculated on AChE and BuChE monomer A that was prepared by the addition of protons, and the protonation states of ionisable groups was set to that corresponding to pH 7.0, temperature 300 K and [NaCl] of 0.1 M using the MOE software (Molecular Operating Environment (MOE), version 2016.08, Chemical Computing Group, Montreal, QC, Canada) and the AMBER 10 force field [50]. The same prepared protein structures were supplied to the Protein Dipole Moments Server [51]. Electrostatic potential maps were generated using a Poisson-Bolzmann solver with a solvent dielectric constant of 80. Potentials were mapped using atomic partial charges from the OPLS_2005 force field in the Maestro software (version 10.5, Schrödinger, Inc.).

## Figures and Tables

**Table 1:** IC_50_ and Hill coefficient of crystal violet (CV).

Protein	IC_50_^1^ (µM)	Hill Coefficient
*m*AChE	2.2 (2.13–2.36)	−2.0 (−2.18–−1.81)
*h*AChE	1.2 (1.16–1.32)	−2.2 (−2.53–−1.91)
*h*BuChE	0.9 (0.87–1.00)	−1.4 (−1.56–−1.29)

^1^ Mean value of at least three determinations, values in parentheses denote the 95% confidence interval. Abbreviations: IC_50_, half maximal inhibitory concentration; *m*AChE, *Mus musculus* AChE; *h*AChE, *Homo sapiens* AChE; *h*BuChE, *Homo sapiens* BuChE.

**Table 2:** The IC_50_ and Hill coefficient for site directed mutants.

Protein	Substitution	IC_50_^1^ (µM)	Factor ^2^	Hill Coefficient
*m*AChE	Wild-type	2.2 (2.13–2.36)		−2.0 (−2.18–−1.81)
	D283A	7.4 (6.85–7.89)	3.4	−1.5 (−1.62–−1.31)
	D283N	6.5 (6.25–6.80)	3.0	−1.8 (−1.92–−1.64)
*h*AChE	Wild-type	1.2 (1.16–1.32)		−2.2 (−2.53–−1.91)
	Y72A	1.4 (1.37–1.49)	1.2	−1.8 (−1.96–−1.73)
	D74A	5.0 (4.70–5.36)	4.2	−1.7 (−1.83–−1.49)
	D74N	5.0 (4.66–5.31)	4.2	−2.1 (−2.44–−1.86)
	N283A	3.7 (3.54–3.78)	3.1	−1.8 (−1.86–−1.68)
	N283D	3.5 (3.38–3.68)	2.9	−1.9 (−2.01–−1.75)
	W286A	4.4 (4.06–4.73)	3.7	−2.1 (−2.49–−1.77)
	Y341A	2.9 (2.76–3.09)	2.4	−2.0 (−2.26–−1.82)

^1^ Mean value of at least three determinations, values in parentheses denote the 95% confidence interval; ^2^ IC_50_ mutant/IC_50_ wild-type*.*

**Table 3:** Calculated dipole moment of wild-type and in silico mutated proteins.

Protein	Substitution	PDB Code	Dipole Moment (Debye) ^1^	Net Charge
*m*AChE	Wild-type	1J06	615 (778)	−8 (−8)
	D283A		605	−7
*h*AChE	Wild-type	4EY4	737 (824)	−9 (−10)
	D74A		659	−8
	Y72A		739	−9
	W286A		738	−9
*Tc*AChE ^2^	Wild-type	1ACE	1744 (1592)	−5 (−9)
*h*BuChE	Wild-type	1P0I	1691 (1655)	8 (5)

^1^ Calculated using the MOE software and The Protein Dipole Moments Server (results within parenthesis) (see experimental details in Section 4.4); ^2^ *Torpedo californica* AChE.

## Abbreviations

The following abbreviations are used in this manuscript:

AChE	Acetylcholinesterase
AChI	Acetylthiocholine iodide
*Aa*AChE	*Aedis aegypti* Acetylcholinesterase
*Ag*AChE	*Anopheles gambiae* Acetylcholinesterase
CV	Crystal Violet, Hexamethylpararosaniline chloride
DFT	Density Functional Theory
*h*AChE	*Homo sapiens* Acetylcholinesterase
*h*BuChE	*Homo sapiens* Butyrylcholinesterase
*m*AChE	*Mus musculus* Acetylcholinesterase
Ortho-7	1,7-heptylene-bis-*N*,*N′*-2-pyridiniumaldoxime dichloride
*Tc*AChE	*Torpedo californica* Acetylcholinesterase
4PL	Four Parameters Logistic

## Appendix A

**Table A1:** Data collection and refinement statistics.

Protein Data Bank Accession Code	5OV9
**Data Collection Statistics**	
Resolution range (Å)	29.1–2.4 (2.486–2.4)
Space group	P 2_1_2_1_2_1_
Unit cell (Å, °)	79.6 113.9 226.7 90 90 90
Total reflections	332,637 (32,579)
Unique reflections	80,635 (7919)
Multiplicity	4.1 (4.1)
Completeness (%)	98.22 (98.00)
Mean I/sigma (I)	16.39 (4.94)
Wilson B-factor	42.99
R_merge_	0.04764 (0.4941)
R_meas_	0.0545 (0.5644)
**Refinement Statistics**	
Reflections used in refinement	79975 (7902)
Reflections used for R-free	1585 (143)
R-work	0.1614 (0.2108)
R-free	0.1989 (0.2587)
CC (work)	0.967 (0.950)
CC (free)	0.944 (0.905)
Number of non-hydrogen atoms	9224
Macromolecules	8334
Ligands	259
Solvent	631
Protein residues	1067
Rmsd from ideal values	
Bond length	0.012
Bond angles	1.35
Ramachandran plot (%)	
Favoured regions	96.40
Allowed regions	3.31
Disallowed regions	0.28

Values for the highest resolution shell is shown in parenthesis.
